# Analysing population-based cancer survival – settling the controversies

**DOI:** 10.1186/s12885-016-2967-9

**Published:** 2016-12-03

**Authors:** Maja Pohar Perme, Jacques Estève, Bernard Rachet

**Affiliations:** 1 Institute of Biostatistics and Medical Informatics, Faculty of Medicine, University of Ljubljana, Vrazov trg 2, 1000 Ljubljana, Slovenia; 2 Université Claude Bernard, Hospices Civils de Lyon, Service de Biostatistique, 162 Avenue Lacassagne, 69003 Lyon, France; 3 Cancer Survival Group, Department of Non-Communicable Disease Epidemiology, London School of Hygiene and Tropical Medicine, London, WC1E 7HT UK

**Keywords:** Relative survival, net survival, Cancer registries, Population-based studies

## Abstract

**Background:**

The relative survival field has seen a lot of development in the last decade, resulting in many different and even opposing suggestions on how to approach the analysis.

**Methods:**

We carefully define and explain the differences between the various measures of survival (overall survival, crude mortality, net survival and relative survival ratio) and study their differences using colon and prostate cancer data extracted from the national population-based cancer registry of Slovenia as well as simulated data.

**Results:**

The colon and prostate cancer data demonstrate clearly that when analysing population-based data, it is useful to split the overall mortality in crude probabilities of dying from cancer and from other causes. Complemented by net survival, it provides a complete picture of cancer survival in a given population. But when comparisons of different populations as defined for example by place or time are of interest, our simulated data demonstrate that net survival is the only measure to be used.

**Conclusions:**

The choice of the method should be done in two steps: first, one should determine the measure of interest and second, one should choose among the methods that estimate that measure consistently.

## Background

For decades, oncologists have shown a great interest in a regular population-based evaluation of their efforts to improve cancer outcome. Clinical trials do not bring any information of the performance of the overall “management” of cancer patients in the general population. Because cancer patients can die from causes other than the studied cancer, Ederer et al. [1] developed an approach to measure “the survival rate so far as the disease under study is concerned” (later called the Ederer I method). Later, Hakulinen proposed a corrected version of Ederer I (Hakulinen method) that explicitly aimed to account for competing causes of death by adjusting for the population survival rate obtained from the life tables, i.e. to estimate net survival as a measure that would be comparable across groups with different population mortality [2]. Both methods have been used until recently by most population-based cancer registries (see e.g. SEER and EUROCARE 1–4). In the same time, another method, often called Ederer II, has also been applied (e.g. EUROCARE 5). Ederer II however reproduces an oversimplification commonly made in conventional survival analysis in a competing risks situation where the deaths of the causes, which are not of primary interest, are simply censored. This method, often referred to as cause-specific survival, is biased when censoring is informative [3], i.e. when the patients with a high hazard of one cause also have a high hazard of the other cause, which is a common situation.

Recently, a specific effort was made to understand the properties of these three relative survival methods, and it has been proven that none of these methods consistently estimates net survival [4]. At the same time, a proposal for a consistent estimator of net survival was made. A debate followed on the suitable methods to be used [5–9].

While most of the work in the past focused on the estimation of net survival, it is clear that being in a competing risks setting, there are several other concepts of interest. Estimation of crude mortality has been proposed by Cronin and Feuer [10] and the usefulness of this measure has been studied by Eloranta et al. [11].

With many methods available and several opposing guidelines given in the literature [7–9, 12], a considerable confusion has arisen in the field, with the result that many studies are not directly comparable since different methodology was used for it. The differences between the ideas underlying the different methods are subtle, so in order to properly understand what should be used and thus resolve the confusion of the field, we believe that, before focusing on any methods, we should first make a clear distinction between the measures and describe their interpretation. This is the main goal of our paper.

The paper is organized as follows: the Methods section reviews four measures of interest and states non-parametric methods that consistently estimate them. The Results section uses colon and prostate cancer data and simulated data to illustrate the differences in interpretation between the measures. The Discussion section comments on the results and often used alternative methods.

## Methods

### Measures of survival and mortality

It is crucial, when planning to analyze the survival experience of cancer patients, to decide upon the goal of the analysis, i.e. which measure of population characteristic we would like to evaluate with our data. We present here four measures most frequently reported in the literature.

- *Overall survival* (*S*_*O*_(*t*)) is the probability that a patient is still alive at a certain time point *t* after the diagnosis. It is directly related to the overall *hazard rate* of dying *λ*_*O*_ – knowing one quantity implies knowing the other. The mathematical relationship between the two is formalized in the following equation:
  1 $$ {S}_O(t)= \exp\ \left( - {\displaystyle \underset{0}{\overset{t}{\int }}}{\lambda}_O(u)du\kern0.5em \right) $$
- *Relative survival ratio* (*S*_*R*_(*t*)) compares the overall survival of the patients to the survival of the cancer-free group with the same demographic structure by calculating the ratio
  2 $$ {S}_R(t)=\frac{S_O(t)}{S_P(t)} $$
  The ratio describes how the observed survival of the patients compares to the survival of a cancer-free group with the same demographic structure.

While overall survival and relative survival ratio do not distinguish between causes of death, the next two concepts assume that the overall hazard *λ*_*O*_ can be split into two additive components - that due to cancer (*λ*_*C*_, often referred to as cause-specific or excess hazard) and that due to other causes (*λ*_*P*_, often referred to as population hazard):

3 $$ {\lambda}_{Oi}(t)={\lambda}_{Pi}(t)+{\lambda}_{Ci}(t) $$

Recognizing that individuals may have different hazards, we use the indicator *i* in the above equation.

- *Cancer-related crude mortality* (*F*_*C*_(*t*)) provides additional information to overall mortality (1 – overall survival). It not only reports the probability of dying up to time *t*, but further splits it into the probability of dying (up to *t*) from cancer *F*_*C*_(*t*) and the probability of dying (up to *t*) from other causes *F*_*P*_(*t*):
  $$ 1-{S}_O(t)={F}_C(t)+{F}_P(t) $$
  The term *F*_*C*_(*t*) is often referred to as the *cancer mortality in the presence of competing risks*, *cumulative cause-specific mortality* (Cronin and Feuer [8]) or simply cancer-related *crude mortality.*
  It can be expressed as
  $$ {F}_C(t)={\displaystyle \underset{0}{\overset{t}{\int }}}{S}_O\left(u-\right){\lambda}_C(u)du $$
  The above formula simply says that in order for a patient to die from cancer at time u, they must have survived both causes until just before that time (*S*_*O*_(*u*−)) and then succumb to hazard *λ*_*C*_. Crude mortality is a cumulative measure –it accumulates deaths at any time *u* up to *t*, hence the integral. Since the overall survival *S*_*0*_ depends on both hazards, the same is true for the crude mortality.
- *Net survival* (*S*_*N*_(*t*)) should be considered if the hazard attributed to cancer is the only hazard of interest, therefore *λ*_*O*_ in equation (1) gets replaced by *λ*_*C*_:
  $$ {S}_N(t)= \exp \left(-{\displaystyle \underset{0}{\overset{t}{\int }}}{\lambda}_C(u)du\right) $$
  Net survival is the only measure that does not depend on the hazard due to other causes and this is the measure to be used when the cancer survival experience of groups with different population mortality is to be compared.
  Crude mortality shall always be lower than net mortality (1 − *S*_*N*_): some patients die from other causes before dying from cancer.
  Using formula (3), net survival of each individual can be written as the ratio of the overall survival probability of this patient and the survival probability of his counterparts in the population. Net survival of a group of size *n* can thus also be interpreted as the average ratio of overall and population survival:
  $$ {S}_N(t)=\frac{1}{n}{\displaystyle \sum_{i=1}^n}\frac{S_{Oi}(t)}{S_{Pi}(t)} $$
  On the other hand, the relative survival ratio (2) of the same group can be written as the ratio of averages:
  $$ {S}_R(t)=\frac{\frac{1}{n}{\displaystyle {\sum}_{i=1}^n}{S}_{Oi}(t)}{\frac{1}{n}{\displaystyle {\sum}_{i=1}^n}{S}_{Pi}(t)} $$
  Obviously the two measures are not mathematically equal and often differ substantially in practice, the extent of the difference depends on the heterogeneity of the individuals with respect to the demographic variables and on the heterogeneity of the individual cancer specific hazards.

### Data settings

Two main settings can be defined according to the availability of the cause of death in the analyzed data. When a cause of death is attributed to each patient, we shall refer to cause-specific data setting. If the cause is not known or unreliable (‘relative survival setting’), indirect information on it can be obtained by merging our data with the general population mortality data available from national statistics, which present the mortality rate (*λ*_*Pi*_) that the patients would experience if they had no cancer (it can be shown that mortality from a specific cancer forms a negligible part of the whole population mortality). Any excess to this mortality can thus be attributed to cancer. Under this setting, relative survival methods, the sole focus of this paper, are applied. Note that the data setting affects the choice of the method of estimation, but not the measure to be estimated.

### Material

To illustrate the properties and interpretation of the measures, we use both real and simulated data. We first analyse data collected by the national population-based cancer registry of Slovenia for patients diagnosed at age 50 to 80 with colon (male and female subjects, *N* = 3184) or prostate (*N* = 2586) cancer between 1990 and 2000, and followed up until the end of 2010 [13]. Time to event or censoring, but not the cause of death, is known for each individual.

We then make a step further from other epidemiological papers (e.g. [6, 12]) by analyzing simulated data, where the truth is known and comparisons thus made easier. We mimic real data to create two data sets A and B with same demographic distributions, and assume that (i) the cohorts were diagnosed in two different calendar years (1990 and 2000) and thus had a different general population mortality rate *λ*_*Pi*_; but (ii) the true, underlying cancer prognosis did not improve between periods A and B, i.e. the cancer-specific hazards *λ*_*Ci*_ are the same. The data sets are large (20000 patients each) to enable clear distinction between the random variation and true differences.

Since our examples are here to highlight the fact that the measures are not equal, we have chosen the simulation parameters so that these differences are rather obvious – the cancer specific hazard in our sample is not homogeneous, but depends rather strongly on age (excess hazard ratio for age equals 1.1), the age of the patients is asymetrically distributed between 50 and 80 with the older patients being more common in the sample.

The information on the hazard of dying from other causes is obtained from the official Slovene life tables split by sex, age and calendar year.

### Statistical methods

Overall survival is estimated using the Kaplan-Meier method - all deaths are considered as events and no information on cause of death or population mortality is needed.

Cronin and Feuer [10] described the methodology for crude mortality estimation using data grouped by fixed-length intervals of follow-up time, we use the continuous time version here.

Net survival is estimated using the recently introduced estimator by Pohar Perme et al. [4] (PP estimator).

Relative survival ratio is estimated using the Ederer I method [1].

## Results

**Fig. 1:**
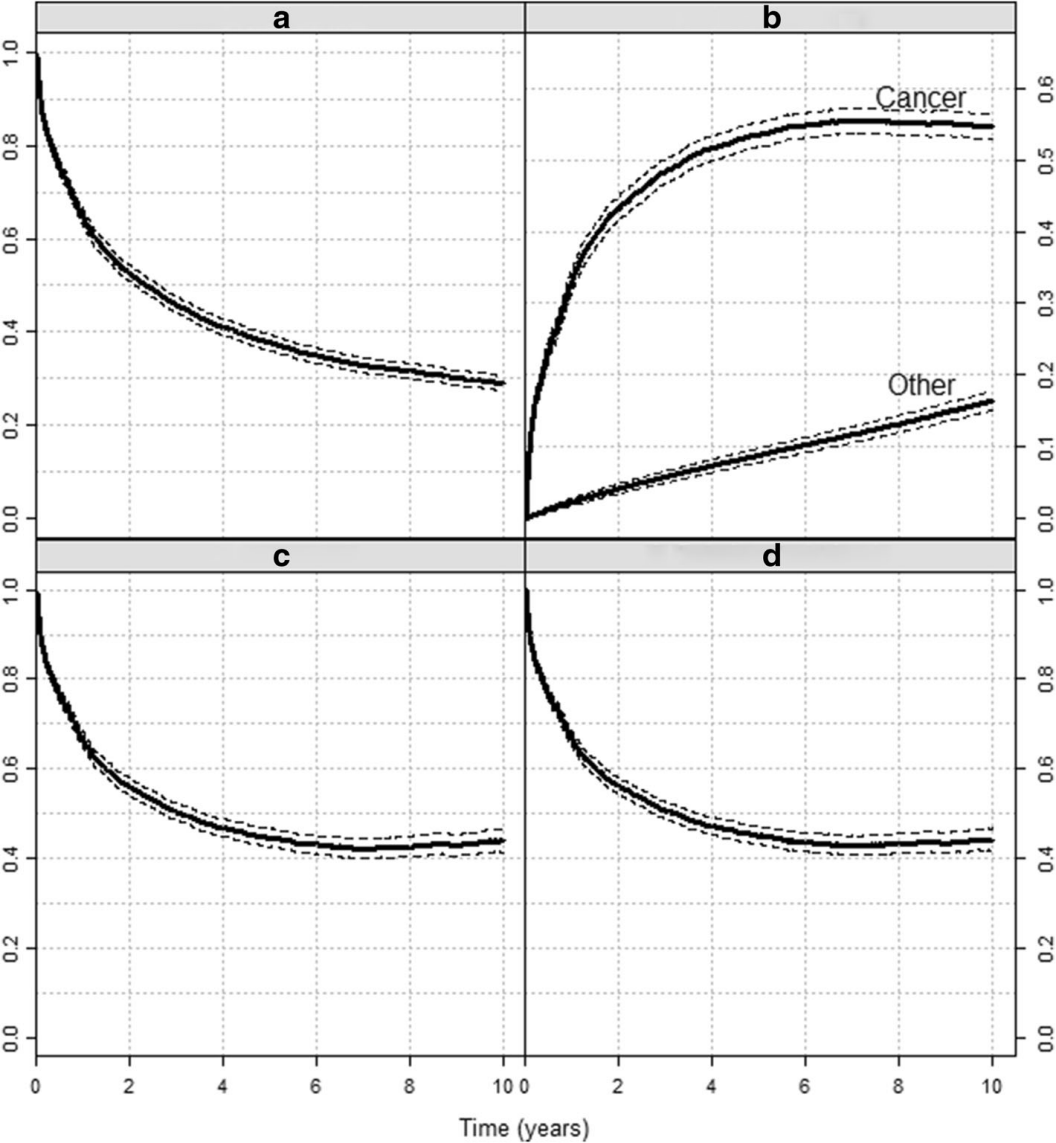
The four measures. Colon cancer survival (1990–2000 patients, Slovenia cancer registry): **a**) Overall survival, **b**) Crude mortality, **c**) Net survival, **d**) Relative survival ratio. Dashed lines denote the 95% confidence intervals. Parts **a**), **b**) and **c**) present probabilities, part **d**) is a ratio

We first consider the cohort of colon cancer patients. Ten-year overall survival equals 0.29 (Fig. 1a). The corresponding 10-year overall mortality of 0.71 (1–0.29) can be split into the probability that a patient dies from colon cancer (0.55) and the probability that a patient dies from other causes (0.16) (Fig. 1b).

Ten-year net survival equals 0.44 (Fig. 1c), i.e. if the patients could only die of cancer, 56% of them would die in the first 10 years after the diagnosis of cancer. This number is slightly higher than the crude probability of dying from the cancer since some patients, in particular older patients, died from other causes before they could die of cancer. Finally, the 10-year relative survival ratio (Fig. 1d) is estimated at 0.44, implying that after 10 years, the survival of our observed cohort is at 44% of the survival of their population counterparts. In this example net and relative survival ratio estimates are equal, but as shown next, this does not have to be the case.

**Fig. 2:**
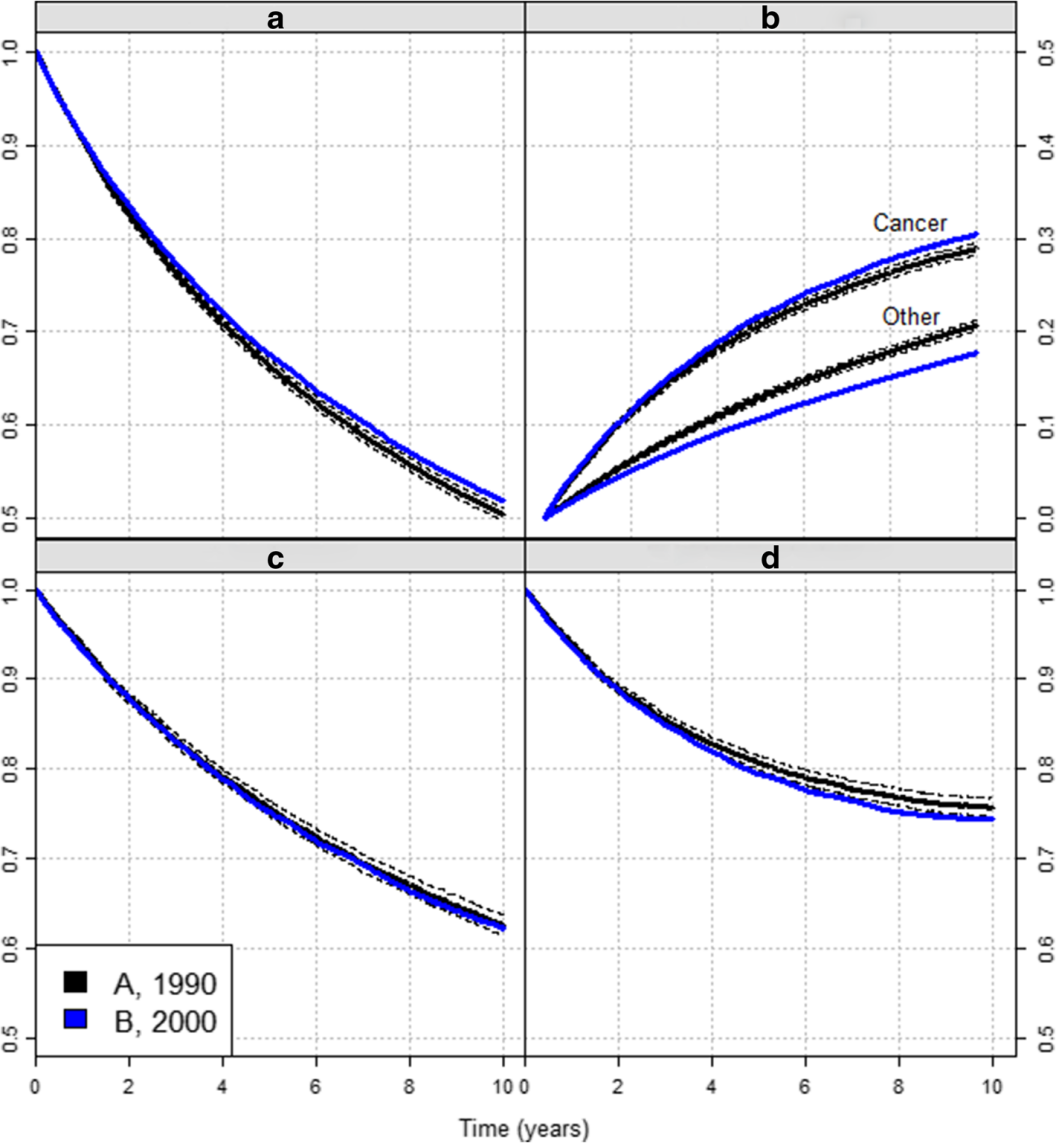
Comparing two cohorts with the different measures. Colon cancer survival (simulated data): **a**) Overall survival, **b**) Crude mortality, **c**) Net survival, **d**) Relative survival ratio. Black curves: cohort A, diagnosed 1990; blue curves: cohort B, diagnosed 2000. Dashed lines denote the 95% confidence intervals for cohort A

Since the cancer patients do not die of cancer only, net survival may seem the least interesting measure. However, its importance becomes obvious when we wish to compare two groups of patients. In our simulated data, the overall survival shows improvement: 10-year survival of the more recently diagnosed cohort B (blue) was 0.52 compared to 0.50 for the earlier cohort A (black, Fig. 2a). The data was simulated so that this improvement in survival is entirely due to improvement in the general population mortality, and this is correctly shown by the equal estimates of net survival in periods A and B (Fig. 2c). By contrast, this information is not obvious from Fig. 2b and d. As expected, the probability of dying from other causes decreased, the decrease after 10 years of follow-up is by 0.03 compared to patients A. However, some of them died from cancer in this period, hence the 0.01 increase in the probability of dying from cancer. Similarly, since the cancer treatment did not improve, but the population survival did, the relative survival ratio of cohort B is lower than that of cohort A (Fig. 2d). In summary, while net survival correctly describes potential change in cancer hazard, neither the crude cancer mortality nor the relative survival ratio can disentangle changes in both components.

**Fig. 3:**
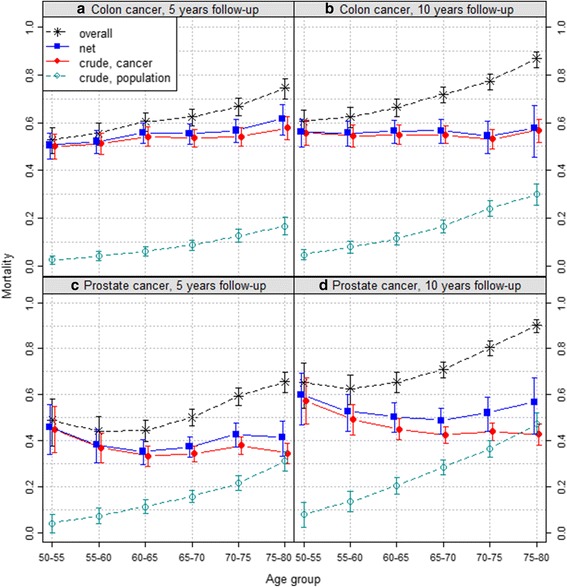
Colon and prostate cancer mortality with respect to age. Colon (*upper row*) and prostate (*lower row*) cancer mortality after 5 (*left*) and 10 (*right*) years (1990–2000 patients, Slovenia cancer registry)

We now turn to comparing the colon and prostate cancer mortality at 5 and 10 years with respect to age (Fig. 3).

Among colon cancer patients, the overall mortality (stars) increases with age. After 10 years, overall mortality rises from 0.61 for the 50–55 years group to 0.87 for the 75–80 group. The same is true for their probability of dying from other causes (empty circles), which is around 25% higher in the oldest group compared to the youngest. On the contrary, their probability of dying from cancer (filled circles) does not change much with age group – after 10 years, 57% of colon cancer patients are estimated to have died from their cancer – and this is also true for net mortality.

By contrast, among prostate cancer patients, the overall mortality is lower in 55–65 than at age 50–55, then increases with age. Net mortality shows a fairly comparable age-related pattern. The probability of dying from the cancer decreases steadily until age 65–70 where it plateaus at 0.43, close to the probability of dying from other causes reached by the oldest age group.

The relative survival ratio is practically the same as net survival in all four graphs and thus not included in the graphs to avoid visual clutter.

## Discussion

Our examples illustrate how the described measures provide fundamentally different, but complementary, information.

This is best illustrated by the 10-year age-related patterns for prostate cancer (Fig. 3d). The U-shape curve of net mortality reflects the worse prognosis of prostate cancer among young and old patients. However, among young patients, net mortality and probability of dying from the cancer are very comparable because these patients mostly died from their cancer, as shown by their very low probability of dying from other causes. By contrast, despite high net mortality denoting a poor prognosis of prostate cancer, less than half of old patients died from their cancer. However, because of the rapid, secular increase in life expectancy, i.e. decrease in probability of dying from other causes, the gap between net and crude cancer mortalities implies that prostate cancer may become an even bigger public health problem in a near future. Indeed, the number of deaths due to prostate cancer may increase dramatically among the elderly patients if net survival of prostate cancer, i.e. its prognosis, does not improve significantly.

The choice of which measure to report is delicate. Crude mortality is more relevant for health policy-makers [11, 14], since it quantifies the actual contribution of the disease to overall mortality. Net survival is the survival probability derived solely from the cancer-specific hazard of dying. Because it is unaffected by differences in mortality from other causes, it is the only measure allowing a proper comparison of different populations according to time, geography or other characteristics.

However, this does not mean that an observed difference in net survival between two groups cannot come from their different demographic structure – for example, if age affects the cancer specific hazard, then the net survival of groups with different age structure is expected to differ and has to be taken into account for example by age-standardization.

Relative survival ratio used to be the main reported measure as it was thought to equal net survival. We believe it may be still appealing as a direct comparison between the overall survival of the patients and the expected survival from the general population.

Two further terms, “relative survival” and “cause-specific survival”, are still often used in the literature as they would describe measures. The former is confusing as it could apply to any measure within the “relative survival setting”. The latter is unfortunate since, while cause-specific mortality aims to estimate the proportion of patients dying from each specific cause, one has to survive all causes to be still alive. We propose to avoid both terms.

Once the measure of interest is determined, one should choose among the methods that estimate it. We describe here the most common alternatives that appear in the field.

One option is to use model-based predictions, i.e. a parametric estimator of the measures. Here, one should keep in mind that the first step in the analysis of survival data is the non-parametric estimation of the survival curve, which gives us a description of the data. This analysis may be followed by modelling the effect of covariates (looking at trends, etc.), which requires modelling assumptions, that can be rather complex and often include interactions. In any case the model specification needs to be checked; a first evaluation can be done simply by drawing the survival curve predicted by the model and check whether it fits well to the non-parametric curve. Therefore, as for any type of analysis, using only the parametric approach to describe the data is not appropriate, but once the model is in this way proven to be acceptable, it is a powerful tool to understand the data in depth and make predictions.

**Fig. 4:**
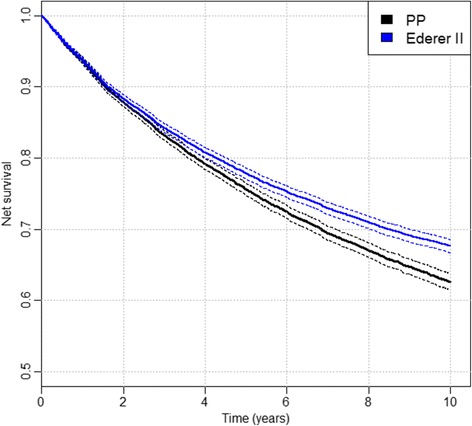
Ederer II and PP estimator. The comparison of Ederer II (*blue solid curve*) and PP estimator (*black solid curve*) on the simulated data set A. The dashed curves present the confidence intervals for each method

A lot of confusion exists about the non-parametric method to use for net survival estimation while the estimation proposals of the above three other measures are quite clear. In the past, the Ederer I or its correction, the Hakulinen method, has been used for estimation. More recently, some authors claim Ederer II should be used [12], but all three methods have been theoretically proven not to be consistent [4]. While the bias in small samples might be hard to discern due to large variation, it still persists in large samples (i.e. the method is not consistent), where variation of the estimates becomes negligible. An example is given in our simulated data set, where at 10 years Ederer II misses the true value by 5% (Fig. 4) and Ederer I misses it for 13% (Fig. 2). This means that, though analysing these same data, one would conclude to significantly different results. Furthermore, a researcher using the Ederer II method on our data would wrongfully conclude that net survival worsened from 1990 to 2000. This example illustrates how the choice of approach can affect results of comparisons between population groups, an issue already raised by Seppå et al. [6].

Fortunately, most publications in the past reported “age-standardized results”, which implies analysis stratified by age and sex, a situation in which the Ederer I, Hakulinen and Ederer II methods give comparable results, provided that the stratification was fine enough. In that case, all patients of each strata have roughly the same hazard and therefore, the relative survival ratio and net survival within the strata become roughly equal. Recent publications have focused on age-stratified Ederer II [8, 9] and have used simulations to support this theoretical fact and to evaluate the size of the bias in practice where the age intervals for stratification are rather wide. Further, the mean square errors of the age-stratified Ederer II were compared to those of the PP method, but the simulations were largely affected by the fact that they included very old patients, for whom very little or no information on long-term net survival is available in the data since their probability of dying of other causes is so high. In such cases, the variance of the PP method becomes very large reflecting that no information is available to estimate net survival of the old patients. On the other hand the variance of the age-stratified Ederer II remains comparatively small, since the estimator relies on the assumption that the younger patients still in the risk set carry the required information. In practice, this assumption may or may not be true, therefore, their variance and bias strongly depended on the simulation parameters and results presented in both papers do not entirely agree. Further work may be needed to clarify these issues.

In practice, a common criterion in choosing a certain method is also the availability of the software. In the relative survival setting, the most recently introduced method is the PP estimator and the inexistence of this method in some of the standard software (e.g. SAS) can be a clear reason for the age-stratified Ederer II method to be used. However, the command availability is not a problem for the R or Stata users. All the methods mentioned in this paper are available in R package relsurv [15], in Stata, the commands stns [16], strs [17] and stnet [18] include the required options. In SEER*Stat the PP estimator is under development.

## Conclusion

In conclusion, we have shown that to provide proper information for cancer control policy all four measures - the overall survival, the relative survival ratio, the crude mortality and the net survival are useful. After determining the measure, we should then choose the method that estimates that measure consistently. When estimating net survival, the traditional methods (Ederer I, Hakulinen, Ederer II) could only be used as a reasonable approximation if they are properly stratified. On the other hand, Ederer I is a perfectly valid estimator of relative survival ratio and, as such, even within heterogeneous population.

## Abbreviations

PP method: Pohar Perme method
